# AI Conversational Agent to Improve Varenicline Adherence: Protocol for a Mixed Methods Feasibility Study

**DOI:** 10.2196/53556

**Published:** 2023-12-11

**Authors:** Nadia Minian, Kamna Mehra, Mackenzie Earle, Sowsan Hafuth, Ryan Ting-A-Kee, Jonathan Rose, Scott Veldhuizen, Laurie Zawertailo, Matt Ratto, Osnat C Melamed, Peter Selby

**Affiliations:** 1 INTREPID Lab Centre for Addiction and Mental Health Toronto, ON Canada; 2 Department of Family and Community Medicine University of Toronto Toronto, ON Canada; 3 Campbell Family Mental Health Research Institute Centre for Addiction and Mental Health Toronto, ON Canada; 4 Department of Pharmacology and Toxicology University of Toronto Toronto, ON Canada; 5 Institute of Medical Sciences University of Toronto Toronto, ON Canada; 6 Edward S Rogers Sr Department of Electrical and Computer Engineering University of Toronto Toronto, ON Canada; 7 Faculty of Information University of Toronto Toronto, ON Canada; 8 Schwartz Reisman Institute for Technology and Society University of Toronto Toronto, ON Canada; 9 Dalla Lana School of Public Health University of Toronto Toronto, ON Canada; 10 Department of Psychiatry University of Toronto Toronto, ON Canada

**Keywords:** evaluation, health bot, medication adherence, smoking cessation, varenicline, artificial intelligence, AI

## Abstract

**Background:**

Varenicline is a pharmacological intervention for tobacco dependence that is safe and effective in facilitating smoking cessation. Enhanced adherence to varenicline augments the probability of prolonged smoking abstinence. However, research has shown that one-third of people who use varenicline are nonadherent by the second week. There is evidence showing that behavioral support helps with medication adherence. We have designed an artificial intelligence (AI) conversational agent or health bot, called “ChatV,” based on evidence of what works as well as what varenicline is, that can provide these supports. ChatV is an evidence-based, patient- and health care provider–informed health bot to improve adherence to varenicline. ChatV has been programmed to provide medication reminders, answer questions about varenicline and smoking cessation, and track medication intake and the number of cigarettes.

**Objective:**

This study aims to explore the feasibility of the ChatV health bot, to examine if it is used as intended, and to determine the appropriateness of proceeding with a randomized controlled trial.

**Methods:**

We will conduct a mixed methods feasibility study where we will pilot-test ChatV with 40 participants. Participants will be provided with a standard 12-week varenicline regimen and access to ChatV. Passive data collection will include adoption measures (how often participants use the chatbot, what features they used, when did they use it, etc). In addition, participants will complete questionnaires (at 1, 4, 8, and 12 weeks) assessing self-reported smoking status and varenicline adherence, as well as questions regarding the acceptability, appropriateness, and usability of the chatbot, and participate in an interview assessing acceptability, appropriateness, fidelity, and adoption. We will use “stop, amend, and go” progression criteria for pilot studies to decide if a randomized controlled trial is a reasonable next step and what modifications are required. A health equity lens will be adopted during participant recruitment and data analysis to understand and address the differences in uptake and use of this digital health solution among diverse sociodemographic groups. The taxonomy of implementation outcomes will be used to assess feasibility, that is, acceptability, appropriateness, fidelity, adoption, and usability. In addition, medication adherence and smoking cessation will be measured to assess the preliminary treatment effect. Interview data will be analyzed using the framework analysis method.

**Results:**

Participant enrollment for the study will begin in January 2024.

**Conclusions:**

By using predetermined progression criteria, the results of this preliminary study will inform the determination of whether to advance toward a larger randomized controlled trial to test the effectiveness of the health bot. Additionally, this study will explore the acceptability, appropriateness, fidelity, adoption, and usability of the health bot. These insights will be instrumental in refining the intervention and the health bot.

**Trial Registration:**

ClinicalTrials.gov NCT05997901; https://classic.clinicaltrials.gov/ct2/show/NCT05997901

**International Registered Report Identifier (IRRID):**

PRR1-10.2196/53556

## Introduction

Medication adherence presents a significant and pressing challenge worldwide across health care systems [1]. This problem spans across various medical conditions, from chronic illnesses such as hypertension, diabetes, and cardiovascular disease to acute infections [2]. Nonadherence to medication regimens can lead to a cascade of problems, including decreased treatment effectiveness, a higher risk of disease progression or complications, medication resistance, increased health care costs, and a reduced overall quality of life for patients [3]. It also places an additional burden on health care providers, who must constantly navigate the complexities of patient adherence to optimize treatment outcomes. Addressing medication adherence is not only a matter of improving individual health but also a vital step toward more efficient health care systems and improving population health.

One notable area where medication adherence could have a significant effect on public health is with smoking cessation medications. Tobacco smoking is not only extremely addictive [4], but it also damages almost every organ in the body [5] and is a leading cause of health inequalities [6]. Despite decades of efforts to reduce the smoking prevalence in Canada [7], over 4 million (15%) Canadians smoked in 2020 [8]. Tobacco use remains the leading cause of preventable death in Canada [9], and following smoking cessation, the risk of developing tobacco-related diseases gradually decreases over time [10].

Varenicline, a partial agonist of the α-4 β-2 nicotinic receptor, is one of the most effective smoking cessation medications associated with a doubling of long-term quitting with 12 weeks of continuous treatment [11]; however, adherence is a major barrier to achieving and maintaining abstinence [12-14]. Among those who take varenicline for smoking cessation, over one-third stop taking the medication by the second week of treatment, and these patients are significantly less likely to quit smoking [15-18]. In addition, several studies that have examined the correlation between sociodemographics and adherence to varenicline have found male sex [15,19], older age [15,19-21], White race [21,22], and higher education to be associated with better adherence [21]. Conversely, 1 study found that greater age was associated with poorer adherence [21].

One of the most effective strategies to improve adherence to medication is tailoring behavioral interventions [23]. Research has also shown that the use of technological solutions is crucial to providing personalized, cost-effective, and evidence-based interventions [24]. One such digital health solution is a web-based program integrating artificial intelligence (AI) conversational agents, or “health bots,” that can be helpful in improving adherence to medication [25]. Health bots have greater functionality compared with applications providing simple text message reminders since they can track medication intake, provide information about side effects, and answer medication-related questions [26,27].

The “ChatV” health bot is an example of such a digital health solution that we developed to improve adherence to the smoking cessation medication varenicline [28]. We used a theory-based, patient-centered approach through the Discover Design Build and Test framework [29] to design the health bot; we conducted a rapid review to explore the barriers and facilitators to varenicline adherence and interviewed 20 patients and 19 health care providers to understand similar barriers and facilitators as well as features to be included in a health bot that may improve varenicline adherence. The data from these sources were analyzed using the capability, opportunity, motivation-behavior change (COM-B) [30] model of behavior change and its associated framework (theoretical domain framework) [31] and taxonomy (behavior change techniques taxonomy) [32] to identify the features to be included in the health bot. We used the Wizard of Oz methodology [33] to build a library of responses that were programmed in the health bot to answer users’ questions. We are currently beta testing the minimal viable product that has the following features: (1) scheduling and receiving reminders for varenicline dosing; (2) setting a smoking quit date; (3) answering questions related to varenicline (eg, guidance for missed doses) and smoking cessation (eg, strategies to reduce cravings); (4) providing information and strategies to manage side effects [12,15,18]; (5) tracking medication use and smoking; and (6) encouraging participants to increase their motivation to maintain medication intake and continue their quit attempts. Throughout the development of the health bot, a health equity lens was adopted to address any disparities in varenicline use and health bot features between different demographic characteristics.

In this protocol, we detail our plans to examine the uptake, usability, acceptability, and appropriateness of ChatV (ie, feasibility) [34] and to determine whether progression to a fully powered randomized controlled trial (RCT) is warranted.

## Methods

### Design

We will conduct a nonrandomized, single-arm feasibility study.

### Participant Recruitment

We will recruit 40 participants. In order to ensure diversity with respect to age, gender, race, and socioeconomic status, we will purposefully recruit participants through a variety of methods, including the research boards of Centre for Addiction and Mental Health (CAMH), where recruitment flyers are posted for patients to explore research opportunities; health care providers at CAMH programs (eg, Smoking Treatment for Ontario Patients [STOP] program, Aboriginal services, and Rainbow services) and partnering organizations (Rainbow Health Ontario); the STOP program’s list of people who are seeking treatment for smoking and who have consented to be contacted for future research studies; and social media and community boards. Research staff members will conduct a prescreening for those who are interested, and if eligible, participants will be enrolled in the study.

### Inclusion Criteria

The inclusion criteria include individuals who are seeking treatment for their tobacco use who smoke 10 or more cigarettes a day, are willing to set a quit date in the next 30 days, are willing to take varenicline for 12 weeks, are aged 18 years, speak and read English, have a smartphone with a data plan, report being committed to answering questions during follow-up, and live in Ontario.

### Exclusion Criteria

The exclusion criteria include having contraindications to varenicline use, being pregnant, planning to become pregnant, breastfeeding, or participating in the co-design phase.

### Ethical Considerations

The CAMH Research Ethics Board (REB #050/2022) has provided approval to conduct the study, and all participants will provide written informed consent before participation. It has been registered at ClinicalTrials.gov (NCT05997901). Any amendments to the protocol are noted, and substantial changes will be reported to the ethics committee. All interested participants will receive an information sheet and a consent form describing the study and providing sufficient information for the participant to make a decision regarding study participation. A research staff member will arrange a personal meeting with the prospective participants and inform them again about all study procedures, benefits, risks, and their rights regarding study participation. Afterward, the consent form is signed by both the participant and the research staff before the participant undertakes any study procedure. The informed consent process is documented in Research Electronic Data Capture (REDCap; Vanderbilt University).

All study-related documents and data will be held in strict confidence and stored on CAMH servers that will follow CAMH policies and procedures to ensure participant privacy and confidentiality. Any data collected as part of the participants’ completion of the electronic consent form will be securely stored in REDCap, a CAMH-approved data collection tool.

In addition to free medication (for 12 weeks), participants will receive an honorarium in the form of a CAD $30 (US $22) gift card after completion of the 4-week follow-up survey and a CAD $40 (US $30) gift card after completion of the 12-week interview.

### Procedures

The research team will inform potential participants about the study and evaluate if they meet the inclusion criteria. If they qualify, participants will receive a consent form and arrange a phone-based discussion to obtain consent. After obtaining consent, participants (n=40) will complete an internet-based questionnaire covering sociodemographic characteristics, nicotine dependence [35], and an adapted scale to measure varenicline adherence self-efficacy [36-38]. After completing the questionnaire, participants will have an internet-based or in-person visit with a health care provider for eligibility confirmation and to obtain a prescription for varenicline for the first 4 weeks (1 tablet [0.5 mg] daily for the first 3 days, 1 tablet [0.5 mg] twice daily for the next 4 days, and 1 tablet [1 mg] twice daily for 3 weeks). In addition, research staff will show participants how to use the health bot on their phones. Medication will be provided in person or, in the case of internet-based contacts, mailed to the participants. A follow-up visit will be scheduled at 2 weeks, where health care providers will assess the participant’s tolerance of varenicline and provide a prescription for the remaining 8 weeks (1 tablet [1 mg] twice daily or a different dose depending on the tolerance of each participant). Any unanticipated problems (ie, adverse events that are unexpected in terms of severity, nature, or frequency; are related or possibly related to participation in the research; and suggest that the research places other research participants at greater risk of harm) will be documented and reported to the research ethics board, and adverse drug reactions will be reported to the market authorization holder (Apotex) as applicable.

Participants will be required to complete surveys at 1, 4, 8, and 12 weeks. When participants join the study, they will be able to opt to respond to these questions using either the health bot or an internet-based platform. Those who do not complete the internet-based or health bot follow-up within the designated time frame will receive a phone call from our research staff. Additionally, after 12 weeks of joining the study, participants will be invited to participate in a 1-hour semistructured phone interview.

### Measures

#### Overview

The taxonomy of implementation outcomes of Proctor et al [39] guides our selection of implementation outcomes (Table 1).

**Table 1:** Summary of measures.

Measures	Survey timeline						Interview		Analytics
	Baseline	Week 1	Week 4	Week 8	Week 12	Week 12		Weeks 1-12	
Demographics	✓								
Adoption						✓		✓	
Acceptability		✓^a^	✓^a^	✓^a^	✓^a^	✓			
Appropriateness		✓^b^	✓^b^	✓^b^	✓^b^	✓			
Fidelity						✓			
Usability		✓^c^	✓^c^	✓^c^	✓^c^	✓			
Medication Adherence		✓^d^	✓^d^	✓^d^	✓^d^	✓			
Smoking Status		✓	✓	✓	✓	✓			

^a^Acceptability of Intervention Measure.

^b^Intervention Appropriateness Measure.

^c^System Usability Scale.

^d^Timeline Followback.

#### Acceptability

Acceptability is the extent to which an innovation is agreeable, palatable, or satisfactory to a stakeholder [39]. In order to measure acceptability, we will use the Acceptability of Intervention Measure (AIM) in the follow-up surveys as well as in the semistructured phone interviews.

#### Appropriateness

Appropriateness is the perceived fit or compatibility of an innovation with a practice setting or context [39]. In this study, appropriateness will be assessed on an individual basis, including factors such as alignment with users’ attitudes, needs, and background, as measured in the 12-week survey with the Intervention Appropriateness Measure (IAM), a validated 4-item intervention appropriateness scale [40], and explored in depth in the semistructured interviews.

#### Fidelity

Fidelity is the extent to which an intervention is used as intended [39]. For this study, in the semistructured interviews, we will investigate the functions of the health bot that participants used; the purposes for which they used it (such as answering questions or seeking support); the frequency of usage; and the quality, specifically whether the health bot provided accurate information.

#### Adoption

Adoption is the intention, decision, or initiation of use for an evidence-based practice, characterizing it at the level of the provider or organization [39]. Since the concept of adoption is closely related to the actual use of systems, researchers investigating behavioral intervention technologies, such as the health bot, have extended this level of analysis to include consumer behavior [41]. In this study, we will measure the adoption of the health bot by analyzing analytics data throughout the 12 weeks that the participant is scheduled to take varenicline. These data will allow us to evaluate the timing (day and time) of user interactions with the health bot, the specific features used, and the duration of engagement. Thus, we will have objective data on the actual adoption of the health bot by each participant. Additionally, during the semistructured interviews, we will probe for participants’ subjective perceptions of their use of the health bot.

#### Usability

Usability will be assessed using the System Usability Scale (SUS) [42]. The SUS is a 10-item measure assessing usability and user satisfaction with technology.

The AIM, IAM, and SUS all have items that are rated on a 5-point Likert scale (1=completely or strongly disagree to 5=completely or strongly agree).

#### Medication Adherence and Smoking Status

Participants will be asked about their smoking status [43] and adherence to varenicline using the Timeline Followback (TLFB) [44] in the surveys collected at 1, 4, 8, and 12 weeks. Participants will report the number of pills taken since the previous assessment. Participants who log their varenicline use in the health bot will be able to refer to these data when filling out the TLFB. We chose the TLFB method for assessing adherence because the 12-week TLFB measure demonstrates a moderate correlation with saliva varenicline levels and is widely regarded as the most practical measure of varenicline adherence [16]. In order to assess the feasibility and accuracy of measuring adherence using TLFB, participants will also be asked to send pictures of their varenicline blister packs at 1, 4, 8, and 12 weeks. This additional measure will also help explore which method to assess adherence should be used for any future research studies (eg, RCT). Smoking abstinence will be defined by a negative response to the dichotomous 7-day point prevalence question, “Have you had a cigarette, even a puff, in the last 7 days?”

### Analysis of Survey Data

We will calculate descriptive statistics, including means, medians, ranges, and SDs, for the demographic data as well as for the AIM, IAM, and analytics data captured by the health bot internal logs. All scores will be analyzed using the appropriate guidelines provided by Weiner et al [40] (acceptability and appropriateness) and Brooke [42] (usability). Consistent with previous studies, adherence will be defined as taking ≥80% of the prescribed varenicline [45-48]. We will not treat adherence as a continuous variable because it is likely to be strongly bimodal and is not necessarily linearly associated with the cessation outcome. We will calculate the percentage of participants who were adherent at week 12 and who reported not having smoked in the last 7 days. These results will serve as progression criteria to determine whether a full-scale RCT is warranted. Although the sample size will be insufficient for meaningful regression analysis, especially given the potentially nonlinear effects of age, we will also test for differences across age and gender groups using 2-tailed *t* tests and chi-square tests. The power for these comparisons will be low; therefore, this analysis will be an exploratory evaluation of large adherence or outcome differences that may be important to future study design, and interpretation will focus on observed differences and CIs.

### Analysis of Semistructured Interviews

We will use framework analysis [49] to analyze the data. Specifically, we will use the implementation outcomes of Proctor et al [39] with an intersectional lens as the a priori framework. Framework analysis is a method used to draw descriptive and explanatory conclusions from qualitative data and includes the following six steps: (1) verbatim transcription of interviews, (2) familiarization of interviews, (3) coding and labeling the data, (4) developing a framework by grouping and categorizing codes, (5) indexing transcripts using the developed framework, and (6) reducing and charting data into a matrix. Interview findings will help us contextualize the findings from the surveys and the information collected from the use of the health bot. For example, we will understand why the health bot was or was not used (adoption); if it was used as intended (fidelity), what about the health bot the participants found agreeable and what they did not (acceptability); what features, if any, aligned with participants’ attitudes, needs, and background (appropriate); and how other features would need to change in order to align with their needs. In addition, we will be able to understand how social identity (eg, age and gender) and power structures (eg, ageism and sexism) played a role in these results. This will provide us with important information to improve the health bot through an equity lens.

### Triangulation of Findings

Once the survey data and interviews are analyzed, we will triangulate the results by listing all the findings on the same page to identify instances where findings from each method align (convergence), provide additional insights on the same topic (complementarity), or seem to conflict with one another (discrepancy or dissonance) [50,51]. Triangulating the data will allow us to contextualize the quantitative findings and explore “intermethod discrepancy” that might lead to a better understanding of how the health bot works and the modifications needed.

### Exploring the Feasibility of the Approaches on Recruitment, Retention, and Data Collection

This study, using “stop, amend, and go” progression criteria for pilot studies [51], will help determine if proceeding with an RCT is a logical next step and whether additional adjustments are necessary. We will only conduct a full-scale RCT if none of the criteria outlined in Table 2 meet the “stop” criterion (unless we see that there are contextual issues that can be modified to overcome the identified problems). If 1 or more of the concepts in Table 2 meet the “amend” criterion, the trial will proceed to an RCT only if the issues can be addressed. If all concepts in Table 2 satisfy the “go” criterion, we will proceed with planning an RCT.

**Table 2:** Criteria to decide if a randomized controlled trial is warranted.

Criteria	Stop	Amend	Go
Patient recruitment	If <30 patients are recruited within 12 months	If 30-40 patients are recruited within 12 months	If 40 patients are recruited within 12 months
Patient retention	If <25% of patients answer the 12-week survey	If 25.1%-79.9% of patients answer the 12-week survey	If ≥80% of patients answer the 12-week survey
Missing data, medication adherence, or smoking status	If >60% of adherence data and smoking status are missing	If 21%-59% of adherence and smoking status data are missing	If ≤20% of adherence and smoking status data are missing
Acceptability and appropriateness	If mean score for the AIM^a^ or IAM^b^ is ≤8	If mean score for the AIM or IAM is 9-12	If mean score for the AIM or IAM is ≥13
Usability	If mean SUS^c^ score is ≤40	If mean SUS score is 41-67	If mean SUS score is ≥68
Adoption	If mean use is ≤ 20 times	If mean use is 21-79 times	If mean use is ≥80 times

^a^AIM: Acceptability of Intervention Measure.

^b^IAM: Intervention Appropriateness Measure.

^c^SUS: System Usability Scale.

## Results

Participant enrollment for the study will begin in January 2024.

## Discussion

Despite the proven effectiveness of varenicline in aiding smoking cessation [52], a significant number of individuals attempting to quit continue to smoke because they struggle with adhering to their varenicline regimen [12-14]. While there are proven strategies to help people adhere to their medications, they have been hard to implement. Digital technology for medication adherence has started to emerge [53,54]; whether these tools can be feasibly implemented and adopted, especially within diverse and disadvantaged populations, remains largely unknown.

In this protocol, we outline the methodology to assess the uptake, usability, acceptability, and appropriateness of ChatV and assist in determining whether it is appropriate to advance to an RCT to gauge the effectiveness of the intervention. This study will address many limitations of previous mobile health trials [55,56], including using an intervention that clearly outlines the behavior-change theory used and the intervention content. In addition, this study provides well-outlined a priori criteria for progressing to an RCT. This will enhance its replicability. Furthermore, this study will provide insights into potential mechanisms underlying the intervention’s impact, shed light on the effectiveness, or ineffectiveness, of its various components, and offer possible explanations.

In summary, the results of this feasibility study will provide much-needed data and insight into the potential implementation of ChatV to augment adherence to smoking cessation medications. In addition, this project has the potential to increase our understanding of how digital tools can improve adherence to other medications in a cost-effective manner.

## Appendix

Multimedia Appendix 1 Peer-review report by the Canadian Institutes of Health Research.

## Abbreviations

AI: artificial intelligence
AIM: Acceptability of Intervention Measure
CAMH: Centre for Addiction and Mental Health
COM-B: capability, opportunity, motivation-behavior change
IAM: Intervention Appropriateness Measure
RCT: randomized controlled trial
REDCap: Research Electronic Data Capture
STOP: Smoking Treatment for Ontario Patients
SUS: System Usability Scale
TLFB: Timeline Followback

## Data Availability

The data sets generated during this study will be available from the corresponding author upon reasonable request.
