# Supplementary material for: AI Conversational Agent to Improve Varenicline Adherence: Protocol for a Mixed Methods Feasibility Study
Source: JMIR Res Protoc. 2023 Dec 11;12:e53556. doi: 10.2196/53556 (PMC10750231; doi:10.2196/53556)
Supplement: Multimedia Appendix 1 [file resprot_v12i1e53556_app1.pdf]

January/Janvier 19, 2022
DCO150GP

Canadian Institutes of Health Research/Instituts de recherche en santé du Canada

Notice of Recommendation/Avis de recommandation

Application Number/Numéro de la demande: 469406
Committee Code/Code du comité: KTR

Applicants/Candidats: Dr. Nadia Minian

With/Avec: Dr. M. Ratto, Prof. J. Rose, Dr. P. Selby, Dr. S. Veldhuizen, Dr. L. Zawertailo

Institution paid/ Établissement payé: Centre for Addiction and Mental Health (Toronto)

Title/Titre: Helping people adhere to their varenicline treatment by co-creating a conversational agent: A feasibility study

Primary Inst./Inst. principal: Health Services and Policy Research
Other Related Inst./ Autres inst. connexes: Cancer Research; Population and Public Health

Competition /Concours: Project Grant
September/Septembre 15, 2021

Number in competition/Nbre de demandes dans le concours: 2014

Peer Review Committee Recommendation, for your information and use/
Recommandation du comité d'examen par les pairs, pour fins d'information et d'utilisation:

**Committee/Comité:**	Knowledge Translation Research
**Number reviewed/ Demandes examinées:**	26
**Application rank within the committee/ Rang de la demande dans le comité:**	3
**Percent Rank within the committee / Rang en pourcentage au sein du comité:**	92%
**Rated / Cote:**	4.44
**Recommended Term/ Durée recommandée:**	2 years/ans 6 months/mois
**Recommended average annual operating amount/ Montant annuel moyen recommandé pour le fonctionnement:**	$146,000
**Recommended equipment amount/ Montant recommandé pour les appareils:**	$0

This document is for information only.
An application rated below 3.50 is ineligible for CIHR funding. For applications rated 3.50 and above, please note that it is the application's rank within the peer review committee that determines whether it is funded, rather than its absolute rating. The final funding decision will be communicated in the Notice of Decision.

Document à titre d'information seulement.
Une demande cotée en dessous de 3,5 n'est pas admissible au financement des IRSC. En ce qui a trait aux demandes cotées 3,50 ou plus, veuillez noter que l'on détermine l'attribution des fonds en fonction du classement obtenu au sein du comité d'examen par les pairs plutôt qu'en fonction du classement absolu. La décision finale relative au financement sera communiquée dans l'Avis de décision.

**Review Type / Type d'évaluation:**	Reviewer 1 / Évaluateur 1
**Name of Applicant / Nom du chercheur:**	Minian, Nadia
**Application No. / Numéro de demande:**	469406
**Agency / Agence:**	CIHR/IRSC
**Competition / Concours:**	Project Grant/Subvention Projet
**Committee / Comité:**	Knowledge Translation Research/Recherche sur l'application des connaissances
**Title / Titre:**	Helping people adhere to their varenicline treatment by co-creating a conversational agent: A feasibility study

Adjudication Criteria/Critères de sélection

Initial Score/Cote Initiale: 4.5

Top/Bottom Selection/Groupe supérieur/inférieur

- ◉ **Top/Groupe supérieur**
- ○ **Bottom/Groupe inférieur**

**Review Type / Type d'évaluation:**	Reviewer 1 / Évaluateur 1
**Name of Applicant / Nom du chercheur:**	Minian, Nadia
**Application No. / Numéro de demande:**	469406
**Agency / Agence:**	CIHR/IRSC
**Competition / Concours:**	Project Grant/Subvention Projet
**Committee / Comité:**	Knowledge Translation Research/Recherche sur l'application des connaissances
**Title / Titre:**	Helping people adhere to their varenicline treatment by co-creating a conversational agent: A feasibility study

Summary of Application/Résumé de la demande:

Varenicline is the most effective smoking cessation medication in Canada requiring a 12 week course. However many patients become non-adherent and fail to complete the course. Intensive support can improve adherence but is very costly. The application aims to:

1.Co-create and develop a chatbot to increase adherence to varenicline.

2.Evaluate the chatbot in terms of:

a) Implementation outcomes, i.e., adoption, acceptability, feasibility, and appropriateness.

b) Medication adherence and smoking cessation success

3.Explore the feasibility of participant recruitment, retention and data collection.

The application will use the Discover, Design/Build and Test Framework to co design the chatbox involving 1) review the literature and conduct interviews with potential users (Discovery Phase), 2) conduct Wizard of Oz testing (Design/Build Phase); 3) Train and test the chatbot (Test Phase). They will recruit (from diverse sources): 20 participants for the interviews, 40 participants for the WoZ, 20 participants for training the chatbot, and 40 participants (different than those recruited for the WoZ) for the feasibility study. They will stratify by age and gender and expect that their recruitment approach will ensure diversity on other factors. WoZ participants will include individuals at different levels of varenicline use.

**Review Type / Type d'évaluation:**	Reviewer 1 / Évaluateur 1
**Name of Applicant / Nom du chercheur:**	Minian, Nadia
**Application No. / Numéro de demande:**	469406
**Agency / Agence:**	CIHR/IRSC
**Competition / Concours:**	Project Grant/Subvention Projet
**Committee / Comité:**	Knowledge Translation Research/Recherche sur l'application des connaissances
**Title / Titre:**	Helping people adhere to their varenicline treatment by co-creating a conversational agent: A feasibility study

Strengths and Weaknesses/Forces et faiblesses:

Strengths

1.Well written and clear application.

2. This is a resubmission. The response to reviewers is very thoughtful and clarifies a number of issues about the practicalities of conducting this research that were missing in the previous version and persuade me about the feasibility and value of the work. The applicants have expanded the team to include a member with more qualitative expertise.

3.The project is led by and EDI who has an impressive track record for someone at their career level. The research team has a nice balance of expertise and experience. Two senior team members have previously worked with Dr Minian

4. There is appropriate use of KT theories and concepts (use of TDF (with the recent intersectionality extension), Taxonomy of Implementation Outcomes, NASSS).

5. They highlight the importance of gender differences to smoking cessation in general (where women less likely to successfully stop) and varenicline (which has greater short term efficacy in women). Gender will be considered during recruitment (to ensure representation) and analysis of the various studies.

6.Clear stop, amend go rules for proceeding to a definitive trial (now including criteria relating to acceptability, appropriateness and usability)

Weaknesses

It would have been nice to have more information about the proposed synthesis (in particular more details about the types of studies that will be included and more methodological detail about how they will code studies using COM-B and BCTs).

**Review Type / Type d'évaluation:**	Reviewer 1 / Évaluateur 1
**Name of Applicant / Nom du chercheur:**	Minian, Nadia
**Application No. / Numéro de demande:**	469406
**Agency / Agence:**	CIHR/IRSC
**Competition / Concours:**	Project Grant/Subvention Projet
**Committee / Comité:**	Knowledge Translation Research/Recherche sur l'application des connaissances
**Title / Titre:**	Helping people adhere to their varenicline treatment by co-creating a conversational agent: A feasibility study

Budget Recommendation/Recommandation budgétaire:

No major concerns.

**Review Type / Type d'évaluation:**	Reviewer 1 / Évaluateur 1
**Name of Applicant / Nom du chercheur:**	Minian, Nadia
**Application No. / Numéro de demande:**	469406
**Agency / Agence:**	CIHR/IRSC
**Competition / Concours:**	Project Grant/Subvention Projet
**Committee / Comité:**	Knowledge Translation Research/Recherche sur l'application des connaissances
**Title / Titre:**	Helping people adhere to their varenicline treatment by co-creating a conversational agent: A feasibility study

Please indicate your appraisal of the integration of sex as a biological variable as a strength, weakness, or not applicable to the proposal./Prière de sélectionner une option pour donner votre évaluation de l'intégration du sexe comme variable biologique en tant que point fort ou point faible de la proposition, ou en tant qu'élément non applicable à la proposition.

- ○ **Strength/Point fort**
- ○ **Weakness/Point faible**
- ◉ **Not applicable/Non applicable**

Please indicate your appraisal of the integration of gender as a socio-cultural determinant of health as a strength, weakness, or not applicable to the proposal./Prière de sélectionner une option pour donner votre évaluation de l'intégration du genre comme déterminant socioculturel de la santé en tant que point fort ou point faible de la proposition, ou en tant qu'élément non applicable à la proposition.

- ◉ **Strength/Point fort**
- ○ **Weakness/Point faible**
- ○ **Not applicable/Non applicable**

**Review Type / Type d'évaluation:**	Reviewer 1 / Évaluateur 1
**Name of Applicant / Nom du chercheur:**	Minian, Nadia
**Application No. / Numéro de demande:**	469406
**Agency / Agence:**	CIHR/IRSC
**Competition / Concours:**	Project Grant/Subvention Projet
**Committee / Comité:**	Knowledge Translation Research/Recherche sur l'application des connaissances
**Title / Titre:**	Helping people adhere to their varenicline treatment by co-creating a conversational agent: A feasibility study

Sex and/or Gender Considerations/Notions de sexe et/ou de genre:

They highlight the importance of gender differences to smoking cessation in general (where women less likely to successfully stop) and varenicline (which has greater short term efficacy in women). Gender will be considered during recruitment (to ensure representation) and analysis of the various studies.

**Review Type / Type d'évaluation:**	Reviewer 2 / Évaluateur 2
**Name of Applicant / Nom du chercheur:**	Minian, Nadia
**Application No. / Numéro de demande:**	469406
**Agency / Agence:**	CIHR/IRSC
**Competition / Concours:**	Project Grant/Subvention Projet
**Committee / Comité:**	Knowledge Translation Research/Recherche sur l'application des connaissances
**Title / Titre:**	Helping people adhere to their varenicline treatment by co-creating a conversational agent: A feasibility study

Adjudication Criteria/Critères de sélection

Initial Score/Cote Initiale: 4.6

Top/Bottom Selection/Groupe supérieur/inférieur

- (●) **Top/Groupe supérieur**
- ( ) **Bottom/Groupe inférieur**

**Review Type / Type d'évaluation:**	Reviewer 2 / Évaluateur 2
**Name of Applicant / Nom du chercheur:**	Minian, Nadia
**Application No. / Numéro de demande:**	469406
**Agency / Agence:**	CIHR/IRSC
**Competition / Concours:**	Project Grant/Subvention Projet
**Committee / Comité:**	Knowledge Translation Research/Recherche sur l'application des connaissances
**Title / Titre:**	Helping people adhere to their varenicline treatment by co-creating a conversational agent: A feasibility study

Summary of Application/Résumé de la demande:

This multi-methods feasibility study, led by an early career researcher, involves the co-creation and evaluation of a theory informed, patient centered chatbot to help people adhere to varenicline treatment (an effective and cost-effective smoking cessation medication that unfortunately, manysmokers do not adhere to largely due to side effects).

The study involves three phases:

•Co-designing the core functionality of a theory-informed chatbot through a literature review on barriers and facilitators to medication adherence, with particular emphasis on varenicline, and mapping the intervention components to behavioural change techniques; interviewing 20 potential users to identify barriers and facilitators of taking varenicline; and conducting Wizard of Oz testing where 40 participants will interact with a chatbot for three days; and involving 20 participants in the testing of a working prototype.

•Evaluating the implementation outcomes; examining medication adherence and smoking cessation success through a pilot test with 40 participants who will be provided with a standard 12-week varenicline regimen. Passive data collection includes adoption measures (how often participants use the chatbot, what features they used and when did they use it); active data collection includes questionnaires at 1, 4, 8, and 12 weeks to assessing self-reported smoking status and varenicline adherence as well as questions regarding acceptability, appropriateness, and usability of the chatbot, and interviews assessing acceptability, appropriateness, fidelity and adoption.

•Exploring the feasibility of participant recruitment, retention and data collection through the ‘Stop, Amend, Go’ progression criteria for pilot studies to decide if a RCT is a reasonable next step.

Assuming the feasibility study warrants it, results will be used to develop a full-scale RCT to test the efficacy of the chatbot, with potential to improving adherence to effective medications that reduce tobacco use and the burden of tobacco related illnesses. There is also potential to improve adherence to other medications.

**Review Type / Type d'évaluation:**	Reviewer 2 / Évaluateur 2
**Name of Applicant / Nom du chercheur:**	Minian, Nadia
**Application No. / Numéro de demande:**	469406
**Agency / Agence:**	CIHR/IRSC
**Competition / Concours:**	Project Grant/Subvention Projet
**Committee / Comité:**	Knowledge Translation Research/Recherche sur l'application des connaissances
**Title / Titre:**	Helping people adhere to their varenicline treatment by co-creating a conversational agent: A feasibility study

Strengths and Weaknesses/Forces et faiblesses:

STRENGTHS

-This is a very well-written proposal by a promising early career researcher with dissemination and implementation expertise and experience, a track record of co-developing interventions with users, and a strong team and institutional environment supporting her.

-The proposal is an excellent build on the team's previous work; particularly interesting is their exploration about why sometimes tools just don’t work (a previous study showed that a decision support tool did not increase the likelihood of practitioners offering an educational alcohol resource, due to physicians’ discomfort, as well as busy-ness and inadequate training)

-The methodology relies heavily on a range of evidence – that the applicant and her team have developed, as well as others. All aspects of the study appear to be evidence-informed; the proposal is well-referenced.

-The mixed methods design is strong, and there is a good emphasis on future implementation outcomes for the future (phase 2); phase 3 – determining whether to move forward or not – is well laid-out.

-The risks and mitigation section is well done.

WEAKNESSES

I found few weaknesses in this application as I feel that previous reviewers’ excellent guidance has been used to improve an already-strong application.

A few things I had questions about as I read:

•To what extent is the chatbot informational vs motivational, and what issues might this raise
•Is there a concern about what happens after 12 weeks on varenicline
•One thing that wasn’t clear to me were the participants in the literature review phase, which the application states will involve “end users and clinicians to redefine the question.” Which end users where these, and which clinicians and how would the latter be recruited?
•The training of the participants by the research assistant on the chatbot is an important step…what will it involve, will it be evaluated, etc. This could shed light on what’s needed in future (i.e., is sending a link to a chatbot enough, or is training required)

**Review Type / Type d'évaluation:**	Reviewer 2 / Évaluateur 2
**Name of Applicant / Nom du chercheur:**	Minian, Nadia
**Application No. / Numéro de demande:**	469406
**Agency / Agence:**	CIHR/IRSC
**Competition / Concours:**	Project Grant/Subvention Projet
**Committee / Comité:**	Knowledge Translation Research/Recherche sur l'application des connaissances
**Title / Titre:**	Helping people adhere to their varenicline treatment by co-creating a conversational agent: A feasibility study

Budget Recommendation/Recommandation budgétaire:

Seems appropriate; advice from previous reviewers was considered

**Review Type / Type d'évaluation:**	Reviewer 2 / Évaluateur 2
**Name of Applicant / Nom du chercheur:**	Minian, Nadia
**Application No. / Numéro de demande:**	469406
**Agency / Agence:**	CIHR/IRSC
**Competition / Concours:**	Project Grant/Subvention Projet
**Committee / Comité:**	Knowledge Translation Research/Recherche sur l'application des connaissances
**Title / Titre:**	Helping people adhere to their varenicline treatment by co-creating a conversational agent: A feasibility study

Please indicate your appraisal of the integration of sex as a biological variable as a strength, weakness, or not applicable to the proposal./Prière de sélectionner une option pour donner votre évaluation de l'intégration du sexe comme variable biologique en tant que point fort ou point faible de la proposition, ou en tant qu'élément non applicable à la proposition.

- ○ **Strength/Point fort**
- ○ **Weakness/Point faible**
- ◉ **Not applicable/Non applicable**

Please indicate your appraisal of the integration of gender as a socio-cultural determinant of health as a strength, weakness, or not applicable to the proposal./Prière de sélectionner une option pour donner votre évaluation de l'intégration du genre comme déterminant socioculturel de la santé en tant que point fort ou point faible de la proposition, ou en tant qu'élément non applicable à la proposition.

- ◉ **Strength/Point fort**
- ○ **Weakness/Point faible**
- ○ **Not applicable/Non applicable**

**Review Type / Type d'évaluation:**	Reviewer 2 / Évaluateur 2
**Name of Applicant / Nom du chercheur:**	Minian, Nadia
**Application No. / Numéro de demande:**	469406
**Agency / Agence:**	CIHR/IRSC
**Competition / Concours:**	Project Grant/Subvention Projet
**Committee / Comité:**	Knowledge Translation Research/Recherche sur l'application des connaissances
**Title / Titre:**	Helping people adhere to their varenicline treatment by co-creating a conversational agent: A feasibility study

Sex and/or Gender Considerations/Notions de sexe et/ou de genre:

The gender focus is strong: the team has taken a gender-based approach in the design, implementation and evaluation of our proposed study, given that gender plays a major role in tobacco addiction and its treatment, as well as in medication adherence. When recruiting participants to co-create and test the chatbot, representation will be sought from people who identify as women (cisgender and transgender), men (cisgender and transgender), non-binary, as well as those of identify as two-spirit and intersex. The team will conduct a gender based analysis to understand how people with different gender identities use the chatbot and benefit from it. The intent is to inform the building of a chatbot in a way that is equitable and ensures people of different gender identities receive appropriate care.

**Review Type / Type d'évaluation:**	Reviewer 3 / Évaluateur 3
**Name of Applicant / Nom du chercheur:**	Minian, Nadia
**Application No. / Numéro de demande:**	469406
**Agency / Agence:**	CIHR/IRSC
**Competition / Concours:**	Project Grant/Subvention Projet
**Committee / Comité:**	Knowledge Translation Research/Recherche sur l'application des connaissances
**Title / Titre:**	Helping people adhere to their varenicline treatment by co-creating a conversational agent: A feasibility study

Adjudication Criteria/Critères de sélection

Initial Score/Cote Initiale: 3.9

Top/Bottom Selection/Groupe supérieur/inférieur

- (●) **Top/Groupe supérieur**
- ( ) **Bottom/Groupe inférieur**

**Review Type / Type d'évaluation:**	Reviewer 3 / Évaluateur 3
**Name of Applicant / Nom du chercheur:**	Minian, Nadia
**Application No. / Numéro de demande:**	469406
**Agency / Agence:**	CIHR/IRSC
**Competition / Concours:**	Project Grant/Subvention Projet
**Committee / Comité:**	Knowledge Translation Research/Recherche sur l'application des connaissances
**Title / Titre:**	Helping people adhere to their varenicline treatment by co-creating a conversational agent: A feasibility study

Summary of Application/Résumé de la demande:

The authors propose to co-design a smoking cessation intervention in the form of an online chatbot to increase adherence to varenicline medication. Methods include a literature review to identify barriers and drivers of medication adherence, a process of mapping intervention components to behaviour change techniques, interviews of potential users to further identify barriers and drivers, 'Wizard of Oz' testing/development of the chatbot tool, and further beta-testing of the developed tool with a separate set of 20 potential participants.

**Review Type / Type d'évaluation:**	Reviewer 3 / Évaluateur 3
**Name of Applicant / Nom du chercheur:**	Minian, Nadia
**Application No. / Numéro de demande:**	469406
**Agency / Agence:**	CIHR/IRSC
**Competition / Concours:**	Project Grant/Subvention Projet
**Committee / Comité:**	Knowledge Translation Research/Recherche sur l'application des connaissances
**Title / Titre:**	Helping people adhere to their varenicline treatment by co-creating a conversational agent: A feasibility study

Strengths and Weaknesses/Forces et faiblesses:

Strengths

This is an interesting project on a high-volume topic by a promising early-career researcher; the work is appropriately early phase, and carefully lays out an approach to determine whether more costly RCTs are warranted.

The proposal is usefully informed by several relevant frameworks (COM-B, TDF, etc), which are generally described well.

Good use of validated measures whenever possible to inform implementation outcomes. Description of qualitative approach and triangulation reasonable. Excellent description of the decision-making process about whether to continue the work to a more formal RCT, but since this seems to be future research I wonder if some of this space could be reclaimed for methods for the current work.

Team is reasonably strong, but relevance of expertise is not always clear; not clear what experience an expert in HCI has with interviewing patients, for example, or who has the KT experience, even though both are reasonably well described in the methods. The expertise may well be there, it just isn't clear in the description of the team. PI is a new investigator; this would be first funding as PI. Has some experience as co-I on tri-council grants. 15 articles published since 2016, all as first author. Just starting as a supervisor.

Weaknesses

Overall, the justification for choosing a chatbot as the delivery approach of course could be stronger. While arguments about COVID placing emphasis on virtual care and need to scale support for adherence are well made, it isn't clear why a chatbot specifically is seen as the best way to address challenges to adherence. What are the mechanisms by which you see this approach as the most promising scalable approach to this issue? To what extent have chatbots been shown to be effective vehicles for behavioural supports of this sort? Some of the mechanisms by which this approach might have its effects (tracking medications, reminders, providing knowledge) are outlined, but the extent to which these address known barriers would strengthen this justification. Do you think the relevant barriers are going to be helped by tracking meds, reminders, etc?

Summary of progress page is heavily skewed towards description of the PI's cv rather than description of progress on the project itself. While description of experience is relevant, how they inform the current project specifically is not clear. Specifying development of relationships, skills, initial literature evaluation, etc could strengthen this section.

User-centered design usually involves identifying a priori usability goals that inform the design process and let you know when to stop testing; these are not provided.

Justification of why a rapid review rather than some other form of review would strengthen.

Justification of the feasibility of recruitment would strengthen; have these recruitment approaches been used in similar studies in the past?

Qualitative methods include an apparent contradiction that needs to be addressed. While there is reference to a consensus process to resolve differences between coders of interviews, there is also discussion of ‘employing inter-rater reliability’; typically it is one or the other, and so if some components of the analysis will enable reliability analysis and others won’t, this should be spelled out.

Authors state that rules for the simple chatbot will come from lit review, interviews, and testing, but that process is not specified. Will this simply be a series of decisions made by a programmer?

Measures include some of Proctor’s taxonomy, but not all; discussion of why the others were excluded would strengthen.

This is a re-submission, and comments indicate considerable responsiveness to many of the comments, but it appears to my (new reviewer) eyes that a couple of the major issues are still outstanding. For example, the KT section should also include planned efforts to engage in KT of the study results. Furthermore, while this project uses several existing KT frameworks to good effect, the case for how this helps advance our understanding of the utility of these frameworks could be more clearly made, as this is part of the mandate of the KTR committee. Previous reviews also pointed out issues with engaging patient partners. The proposal mentions the SPOR framework, but there is no mention of patient study partners. The decision not to include patient partners as part of the study team should be justified, as this seems a clear situation where patient involvement in the design of this work would be beneficial. Letters of support from relevant patient organisations would strengthen.

**Review Type / Type d'évaluation:**	Reviewer 3 / Évaluateur 3
**Name of Applicant / Nom du chercheur:**	Minian, Nadia
**Application No. / Numéro de demande:**	469406
**Agency / Agence:**	CIHR/IRSC
**Competition / Concours:**	Project Grant/Subvention Projet
**Committee / Comité:**	Knowledge Translation Research/Recherche sur l'application des connaissances
**Title / Titre:**	Helping people adhere to their varenicline treatment by co-creating a conversational agent: A feasibility study

Budget Recommendation/Recommandation budgétaire:

Seems appropriate.

**Review Type / Type d'évaluation:**	Reviewer 3 / Évaluateur 3
**Name of Applicant / Nom du chercheur:**	Minian, Nadia
**Application No. / Numéro de demande:**	469406
**Agency / Agence:**	CIHR/IRSC
**Competition / Concours:**	Project Grant/Subvention Projet
**Committee / Comité:**	Knowledge Translation Research/Recherche sur l'application des connaissances
**Title / Titre:**	Helping people adhere to their varenicline treatment by co-creating a conversational agent: A feasibility study

Please indicate your appraisal of the integration of sex as a biological variable as a strength, weakness, or not applicable to the proposal./Prière de sélectionner une option pour donner votre évaluation de l'intégration du sexe comme variable biologique en tant que point fort ou point faible de la proposition, ou en tant qu'élément non applicable à la proposition.

- ◯ **Strength/Point fort**
- ◯ **Weakness/Point faible**
- ◉ **Not applicable/Non applicable**

Please indicate your appraisal of the integration of gender as a socio-cultural determinant of health as a strength, weakness, or not applicable to the proposal./Prière de sélectionner une option pour donner votre évaluation de l'intégration du genre comme déterminant socioculturel de la santé en tant que point fort ou point faible de la proposition, ou en tant qu'élément non applicable à la proposition.

- ◉ **Strength/Point fort**
- ◯ **Weakness/Point faible**
- ◯ **Not applicable/Non applicable**

**Review Type / Type d'évaluation:**	Reviewer 3 / Évaluateur 3
**Name of Applicant / Nom du chercheur:**	Minian, Nadia
**Application No. / Numéro de demande:**	469406
**Agency / Agence:**	CIHR/IRSC
**Competition / Concours:**	Project Grant/Subvention Projet
**Committee / Comité:**	Knowledge Translation Research/Recherche sur l'application des connaissances
**Title / Titre:**	Helping people adhere to their varenicline treatment by co-creating a conversational agent: A feasibility study

Sex and/or Gender Considerations/Notions de sexe et/ou de genre:

Lack of information on feasibility of recruitment is exacerbated by plans to stratify by gender, age, etc., so more details would strengthen. Good use of SAGER guidelines, and use of intersectional lens is a strength.

Review Type/Type d'évaluation: SO Notes /Notes de l'agent scientifique
Name of Applicant/Nom du chercheur: Minian, Nadia
Application No./Numéro de demande: 469406
Agency/Agence: CIHR/IRSC
Competition/Concours: 2021-09-15 Project Grant/Subvention Projet
Committee/Comité: Knowledge Translation Research/Recherche sur l'application des connaissances
Title/Titre: Helping people adhere to their varenicline treatment by co-creating a conversational agent: A feasibility study

---

Assessment/Évaluation:

Strengths (including SGBA considerations):

This is the second resubmission to this committee, and the applicant's response to the last review was very thoughtful and responsive, and the were able to clarify a number of issues previous reviewers raised.

This was a well written and clear application.

The research team is strong and capable. They expanded the team to include more qualitative expertise. The applicant is an early career researcher with an impressive track record. She will be supported by senior people who she has worked with previously.

The application uses KT theories and concepts (COM-B, TDF, etc) appropriately.

The methodology relies heavily on a range of evidence. Each aspect of the study is evidence-informed. The applicants were cautious about stating that a chatbot might not be a reasonable solution, but there are reasons why it is worth exploring.

Their mixed methods design is strong. There is good emphasis on future implementation outcomes for phase 2. There are clear stop, amend, and go rules for proceeding to a definitive trial.

The risk and mitigation section is well laid out.

This application is very strong in terms of SGBA – the team has taken a gender-based approach in their design, with gender being considered during recruitment and analyses.

Weaknesses (including SGBA considerations):

More information on the proposed synthesis (e.g., the types of studies that will be included and more methodological detail) would be valuable.

To what extent is the chatbot informational vs motivational?

Review Type/Type d'évaluation: SO Notes /Notes de l'agent scientifique
Name of Applicant/Nom du chercheur: Minian, Nadia
Application No./Numéro de demande: 469406
Agency/Agence: CIHR/IRSC
Competition/Concours: 2021-09-15 Project Grant/Subvention Projet
Committee/Comité: Knowledge Translation Research/Recherche sur l'application des connaissances
Title/Titre: Helping people adhere to their varenicline treatment by co-creating a conversational agent: A feasibility study

Assessment/Évaluation:

The training of chatbot by RAs: this training could be better described. It would also be valuable to study how this training will happen in practice.

Recruitment and feasibility: how will they reach all the populations they intend to recruit and their recruitment targets for each group? Will this be a challenge? However, the committee noted this is a feasibility study, which will address whether or not this is feasible.

They cite references for and state gender differences in use of the medicine of choice but the reviews they reference are on sex differences, not gender differences. (REFS 8-10)

The justification for choosing a chatbot could be stronger. Although they talk about mechanisms by which a chatbot may be valuable, it is not clear whether these are the actual problems.

Description of user-centred design usually involves *a priori* goals. They didn’t lay these goals out.

Qualitative design: the applicants discuss both consensus and inter-rater reliability. Which is it?

They mention the SPOR Patient Engagement framework, but it was not clear how this framework in particular would be used to engage patient partners in this study.

The applicants talk about scale-up at times. While the intervention needs to be shown effective before scale up, at this early stage, they could certainly begin to explore indicators of scalability.

While there are clear stop, amend, and go rules for proceeding to a definitive trial, they still feel a bit generic. E.g., they have not specified their criteria with regard to transgender or cisgender participants.

Budget:

No major budget concerns.

**Review Type/Type d'évaluation:**	SO Notes /Notes de l'agent scientifique
**Name of Applicant/Nom du chercheur:**	Minian, Nadia
**Application No./Numéro de demande:**	469406
**Agency/Agence:**	CIHR/IRSC
**Competition/Concours:**	2021-09-15 Project Grant/Subvention Projet
**Committee/Comité:**	Knowledge Translation Research/Recherche sur l'application des connaissances
**Title/Titre:**	Helping people adhere to their varenicline treatment by co-creating a conversational agent: A feasibility study

Assessment/Évaluation:

****

Note: The final rating of the application, provided in the Notice of Recommendation (NOR) and Notice of Decision (NOD), is the averaged rating of the peer review committee members following the discussion of the application during the committee meeting, and therefore may differ from the ratings provided by the assigned reviewers in their respective reviews.

Remarque : La cote définitive de la demande, qui apparaît dans l'avis de recommandation et l'avis de décision, représente la moyenne des cotes accordées par les membres du comité d'évaluation par les pairs après avoir débattu de la demande à la réunion du comité. Elle peut donc différer de celle donnée par les évaluateurs dans leur évaluation respective.
